# Origin and evolutionary history of *Populus* (Salicaceae): Further insights based on time divergence and biogeographic analysis

**DOI:** 10.3389/fpls.2022.1031087

**Published:** 2022-12-16

**Authors:** Xia Liu, Zhaoshan Wang, Wei Wang, Qinqin Huang, Yanfei Zeng, Yu Jin, Honglei Li, Shuhui Du, Jianguo Zhang

**Affiliations:** ^1^ College of Landscape Architecture and Life Science, Chongqing University of Arts and Sciences, Chongqing, China; ^2^ State Key Laboratory of Tree Genetics and Breeding, Key Laboratory of Silviculture of the State Forestry Administration, Research Institute of Forestry, Chinese Academy of Forestry, Beijing, China; ^3^ State Key Laboratory of Systematic and Evolutionary Botany, Institute of Botany, Chinese Academy of Sciences, Beijing, China; ^4^ College of Biology and Food Engineering, Chongqing Three Gorges University, Chongqing, China; ^5^ Henan Academy of Forestry/Quality Testing Center for Forestry Products of National and Grassland Administration, Zhengzhou, China; ^6^ Forestry College, Shanxi Agricultural University, Shanxi, China

**Keywords:** biogeography, divergence time, molecular phylogeny, *Populus*, bering land bridge

## Abstract

**Introduction:**

*Populus* (Salicaceae) species harbour rich biodiversity and are widely distributed throughout the Northern Hemisphere. However, the origin and biogeography of *Populus* remain poorly understood.

**Methods:**

We infer the divergence times and the historical biogeography of the genus *Populus* through phylogenetic analysis of 34 chloroplast fragments based on a large sample.

**Results and Discussion:**

Eurasia is the likely location of the early divergences of Salicaceae after the Cretaceous-Paleogene (K-Pg) mass extinction, followed by recurrent spread to the remainder of the Old World and the New World beginning in the Eocene; the extant *Populus* species began to diversity during the early Oligocene (approximately 27.24 Ma), climate changes during the Oligocene may have facilitated the diversification of modern poplar species; three separate lineages of *Populus* from Eurasia colonized North America in the Cenozoic via the Bering Land Bridges (BLB); We hypothesize that the present day disjunction in *Populus* can be explained by two scenarios: (i) *Populus* likely originated in Eurasia and subsequently colonized other regions, including North America; and (ii) the fact that the ancestor of the genus *Populus* that was once widely distributed in the Northern Hemisphere and eventually wiped out due to the higher extinction rates in North America, similar to the African Rand flora. We hypothesize that disparities in extinction across the evolutionary history of *Populus* in different regions shape the modern biogeography of *Populus*. Further studies with dense sampling and more evidence are required to test these hypotheses. Our research underscores the significance of combining phylogenetic analyses with biogeographic interpretations to enhance our knowledge of the origin, divergence, and distribution of biodiversity in temperate plant floras.

## 1 Introduction

A primary objective of study in the biological sciences is to achieve an understanding of the evolutionary history and biogeographic processes of species that are responsible for shaping their global patterns of variety. In the Northern Hemisphere, one of the best examples of a biogeographic pattern is known as the intercontinental disjunction. Some more traditional disjunct distribution patterns include northern temperate disjunctions (Milne, 2006). Commonly observed disjunctions are Madrean-Tethyan (Wen and Ickert-Bond, 2009) and the eastern Asian-Northeast American disjunctions (Wen, 1999; Wen, 2001; Hao et al., 2015). However, East Asian–Northwest American disjunctions (Wen, 1999; Nie et al., 2005; Zhou et al., 2012) have only infrequently been investigated utilizing current phylogenetic and biogeographic methods. Although the fact that numerous hypotheses regarding the causes of particular kinds of disjunctions have been put forward in research conducted by a variety of different groups, it has been determined that climate changes that occurred during the Cenozoic and two major migration routes, the Bering Land Bridge (BLB) and the North Atlantic Land Bridges (NALB) (Tiffney, 1985a; Tiffney, 1985b; Donoghue et al., 2001; Sanmartín et al., 2001; Zhang et al., 2013), are thought to be the main drivers for the biogeographic patterns across the different regions. Given the intricate biotic responses to these abiotic events that have been observed in the Northern Hemisphere, there is still much to learn about the historical development of disjunct distribution patterns and the underlying evolutionary mechanisms of species diversification. A deeper understanding of the evolution and geographic diversity of the Northern Hemisphere flora can be gained through large-scale biogeographic investigations of plant taxa that exhibit different intercontinental disjunctions (Hao et al., 2015).

Species of the genus *Populus* (Salicaceae), often known as poplars, cottonwoods and aspens, are widely distributed throughout the Northern Hemisphere from subtropical and boreal forests (Eckenwalder, 1996a; Cronk, 2005), and China has unique ecological conditions that make it an important natural distribution and diversity centre for poplars (Wan et al., 2013). A recent taxonomic study classified the 29 species in the genus *Populus* into six divisions (*Abaso*, *Aigeiros*, *Leucoides*, *Populus*, *Tacamahaca*, and *Turanga*) defined by the presence or absence of significant hybridization barriers (Eckenwalder, 1996a; Eckenwalder, 1996b). Most poplars exhibit a high level of ecological plasticity, with diverse adaptations and large population sizes. Section *Abaso*, with a single species *P. mexicana* Wesmael, and section *Turanga*, with three members, *P. euphratica* Olivier, *P. pruinosa* Schrenk and *P. ilicifolia* (Engl.) Rouleau, are restricted to warmer areas; species from sections *Aigeros* and *Tacamahaca* are found in riparian habitats; species from section *Populus* are found in drier areas; and most from section *Leucoides* live in swamps (Karrenberg et al., 2002). Additionally, poplars are known for their fast growth rates, extensive production of wood, along with their small genome sizes (Cronk, 2005; Hamzeh et al., 2006). Thus, poplars have been used as model organisms for many tree-related studies (Jansson and Douglas, 2007; Kuzovkina and Vietto, 2014). *Populus* is a genus that has a large number of species, has extensive records mainly across the Northern Hemisphere, and has unmistakable interspecific affinities (Liu et al., 2017). However, there are no phylogenetically based estimations of divergence times, and the biogeography of *Populus* is poorly understood. Currently, most research on the origin and evolution of *Populus* has been based exclusively on the fossil record (Manchester et al., 1986; Boucher et al., 2003; Manchester et al., 2006). Lineages and their biogeographic distributions have been mapped with the use of molecular phylogenies, and significant geological and ecological events have been more precisely dated using molecular clock, with fossil records serving as a calibrator for gene phylogenies (Berbee and Taylor, 2010).

**Figure 1:**
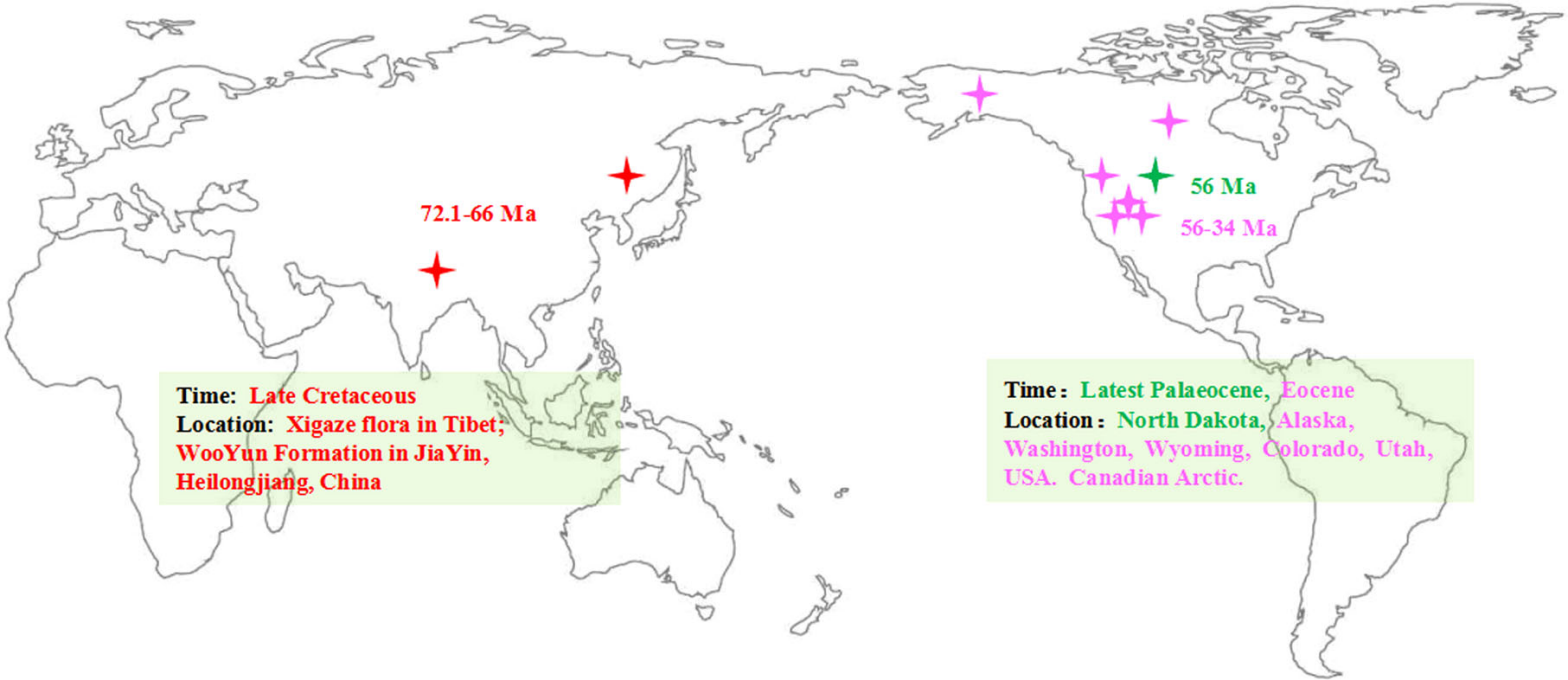
Map of the earlier fossil distribution of the genus Populus (the map adapt from Hao et al., 2015). The asterisk represented the location of the fossils.

To provide a timeline of a group’s evolutionary and biogeographic history, it is essential to have a solid understanding of that group’s fossil history (Helmstetter et al., 2019). The fossil records of *Populus* are extensive and common throughout the history of evolution (**Supplementary Table S1**). In China, the terrestrial strata of the Cretaceous and Tertiary are well developed and widely distributed, and abundant plant fossils have been preserved. Fossils that can be reliably attributed to *P. latior* Ai.Braum (Guo, 1975; Guo, 1983; Guo, 1985) have been dated to the late Cretaceous, corresponding to the Xigaze flora in Tibet, and another Cretaceous (Maestrichtian-Danian) fossil (72.1–66 million years ago, Ma) has been obtained from the Wuyun Formation in Jiayin, Heilongjiang (Guo, 1975; Guo, 1983; Guo, 1985; Tao and Xiong, 1986). According to fossil evidence, in the Cretaceous, *Populus* was widely dispersed in the Northern Hemisphere (**Figure 1**; **Supplementary Table S1**). In addition, all outgroups of Salicaceae and the greatest extant diversity of poplar are only found in Asia (Boucher et al., 2003; Cronk, 2005), but the basal group of Salicaceae and its fossils have not been found in North America. In the past few decades, robust phylogeny-based biogeography combined with molecular dating and ancestral area reconstruction methods has been frequently employed to trace the evolutionary and biogeographic history of species and factors driving the ranges of expansion of many plants (Riddle et al., 2008; Yu et al., 2010; Ali et al., 2012; Portelli and Quinteros, 2018). Nevertheless, the genesis and evolution of modern *Populus* biodiversity remain poorly understood. Here, we first evaluated divergence periods and recreated ancestral ranges to infer the historical processes that led to the modern global distribution of the genus *Populus*.

## 2 Materials and methods

### 2.1 Taxon sampling and phylogenetic analyses

Detailed procedures for DNA extraction, PCR, sequencing, and phylogenetic analysis, are provided (Liu et al., 2017). Briefly, we collected samples from all 34 species in the genus *Populus*, representing all six sections of the genus according to the taxonomy system of Eckenwalder (1996a) and Wu (1999); four species in the genus *Salix*, and three species of related genera of Salicaceae were sampled as outgroups for phylogenetic analyses. Details of voucher information and GenBank numbers for all samples (including the new species) are mentioned in **Supplementary Table S2**. In addition, the chloroplast (cp) fragment molecular dataset was used to reconstitute *Populus* species-level trees in this study, which were then utilized for divergence time calculation.

### 2.2 Phylogenetic inference and divergence time estimation

The sequences were aligned using ClustalX v1.83 (Thompson et al., 1997) and further adjusted manually in BioEdit (Hall, 1999). A number of loci or regions that were unable to be amplified were regarded as missing data. The 34 cpDNA fragments were combined into a single dataset for phylogenetic inference using maximum parsimony (MP), maximum likelihood (ML), and Bayesian inference (BI) with PAUP* v4.0b10 (Swofford, 2003), PhyML v2.4.4 (Guindon and Gascuel, 2003) and MrBayes v3.1.2 (Ronquist and Huelsenbeck, 2003), respectively.

The earliest fossil has been dated to 72.1–66 Ma (Guo, 1975, 1983, 1985; Tao and Xiong, 1986), this means that the most conservative minimum for the genus origin is 66 Ma, but it does not mean that 72.1 Ma is the conservative maximum, as *Populus* may have existed well before 72.1 Ma (though presently without such older fossils identified). A reasonable conservative maximum is 93 Ma, according to Davis et al. (2005) and the source (https://www.mobot.org/mobot/research/apweb/orders/malpighialeswebnew.htm ). Therefore, we utilized internal calibration to establish a divergence time of 66-93 Ma between *Populus* and *Salix*. As in prior research, a lognormal distribution with soft limits and confidence intervals was used by calibrating the means and standard deviations, allowing for the incorporation of error around the projected dates and stratigraphic assignment of the fossils themselves (Su et al., 2017). Divergence times were assessed utilizing BEAST v1.8.3 (Drummond et al., 2012) under a relaxed uncorrelated lognormal clock model utilizing the GTR+I+G substitution model evaluated by jModeltest v3.0.4 (Posada, 2008), birth-death tree prior, the relaxed lognormal clock model, and bootstrap rates using a calibrated rate ranging from 5.10e-4-9.19e-4 substitutions/site/myr (million years) based on Lanfear et al. (2013). Samples were taken every 5,000 generations from three independent runs of 500 million generations. After that, the results of the three separate runs were merged utilizing LogCombiner v2.0 (Drummond and Rambaut, 2007), and the first 10% of the samples were discarded as burn-in. Calculating the effective sample sizes of the parameters utilizing Tracer v1.7 (Rambaut et al., 2018) allowed us to evaluate convergence and ensure sufficient sampling. The values of the effective sample size (ESS) for all of the parameters exceeded 200. Trees were summarized as a maximum clade credibility (MCC) tree (maximum posterior probabilities) utilizing Tree Annotator v2.0 (Drummond and Rambaut, 2007), and the mean ages, 95% highest posterior density (HPD) intervals, posterior probabilities and substitution rates were calculated for each node. FigTree v1.4.2 (Rambaut, 2014) was utilized to depict the divergence tree.

### 2.3 Ancestral area reconstruction

Ancestral areas were reconstituted utilizing RASP v4.2 (Yu et al., 2020): (1) Statistical Dispersal-Violation Analysis (S-DIVA), which explicitly uses a whole posterior distribution of trees to account for both phylogenetic uncertainty and uncertainty in ancestral states, and (2) a Dispersal-Extinction-Cladogenesis (DEC) model in Lagrange analysis (Ree and Smith, 2008), which requires data on a single ultrametric-dated phylogeny and distributional information of extant species. Not only does the Lagrange analysis estimate the ancestral states, but it also calculates the probabilities of the areas at each node (Hao et al., 2015). These are helpful methodological instruments that eliminate the need for arbitrarily defined areas. The species ranges of the genus *Populus* were separated into 5 regions to accomplish biogeographic analysis. These regions were separated as follows: (A) Asia and surrounding areas, (B) Europe, (C) North America (including Mexico), (D) Central Asia, and (E) North Africa. Ancestral area reconstruction was performed using a condensed tree generated from the BEAST analysis. The biogeographical data were coded in accordance with the range of extant poplar species that were selected for this study.

## 3 Results

### 3.1 Phylogenetic inference

**Figure 2:**
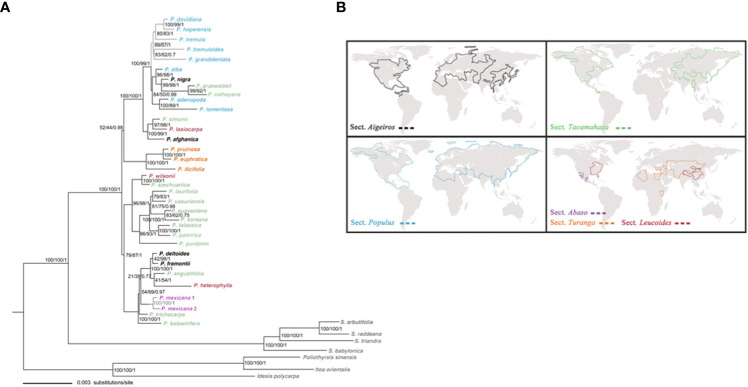
**(A)** The ML tree of *Populus* and outgroups based on combined 34 plastid fragments. Numbers above branches indicate bootstrap values for the MP and ML analyses, and Bayesian posterior probabilities, respectively. **(B)** The geographical distributions of six sections of genus *Populus* is modified from Wang et al. (2020).

We compiled the dataset by sampling the greatest number of *Populus* species to conduct phylogenetic analysis. The concatenated and aligned molecular datasets of 34 plastid fragments consisted of 36,031 base pairs (excluding outgroups). In the ML, the evolutionary model that provided the best match for the combined cpDNA datasets was the GTR+I+G model, while the TIM2+I+G model provided the best fit for the Bayesian analysis. Three methods, including MP, ML, and BI, utilized phylogenetic tree construction yielded similar topology. The chloroplast phylogenies also support *Populus* monophyly in the present research, confirming the conclusions of the vast majority of prior molecular phylogenetic investigations (Cervera et al., 2005; Liu et al., 2017; Wang et al., 2020). *Populus* species may be categorized into three largely supported clades that highly correspond to geographic distributions based on the combined cpDNA dataset analyses (**Figure 2B**). Clade I comprised all Eurasian species, with the exception of two species from North America (*P. tremuloides* and *P. grandidentata*) and one North African species (*P. ilicifolia*). Clade II comprised all the East Asian species that were well supported (96% MP, 98% BS, 1.00 BPP). These Asian species were all *Tacamahaca* species, but the extant *Populus* species in North America have differentiated into *Aigeiros*, *Tacamahaca* and *Leucoides*. Clade III consisted of the remaining seven North American species (**Figure 2A**). Our phylogenetic results obtained from the combined chloroplast dataset had high resolution.

### 3.2 Divergence time estimates

**Figure 3:**
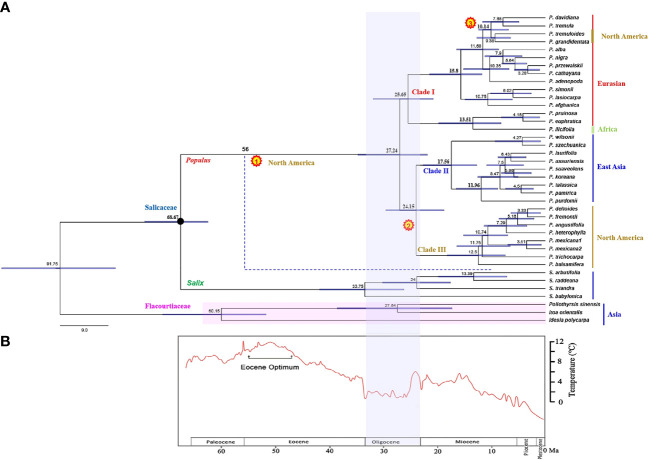
**(A)** Divergence time of *Populus* estimated from combined chloroplast DNA fragments. The black pie charts indicate the nodes calibrated by the fossils (66–93 Ma). The divergence time estimates shown on the nodes are median ages. Numbers in yellow pie charts show three times independently reached into North America events, respectively. The blue dotted line indicates an extinct branch that colonization of North America. The solid line of color next to the taxa names represent the different distribution of species. **(B)** The depiction of climate change past 60 million years before present is modified from Zachos et al. (2001).

Two hybrids, *P. hopeiensis* and *P. tomentosa*, were excluded in estimating the divergence time, because we mainly focused on divergence at the species level of *Populus*. A total of 39 sequences from the combined chloroplast dataset were used for divergence time estimation. In fact, excluding the two hybrids did not significantly affect the overall divergence-age estimates (**Supplementary Figure S1**), hence they were removed to improve the overall topological support values instead. Timetree revealed estimated divergence times between Salicaceae and Flacourtiaceae (91.75 Ma, 95% HPD: 81.74–115.79) and between *Populus* and *Salix* (68.67 Ma, 95% HPD: 63.73–75.05) (**Figure 3A**). The diversification of modern *Populus* species occurred in the late Oligocene at 27.24 Ma (95% HPD: 21.64–35.18). The divergence time estimation of Clade I taxa was 25.65 Ma (95% HPD: 19.87–31.96), which was slightly earlier than the divergence time of the Clade II and Clade III groups, which both diverged at 24.15 Ma (95% HPD: 19.03–29.12). Clade II comprises species from East Asia that first diverged at 17.56 Ma (95% HPD: 13.27–22.63). The divergence time corresponding to the rapid diversification of this clade was 11.96 Ma (95% HPD: 9.14–16.83). Clade III, a North American branch with a shared ancestor according to the plastid data, is the result of differentiation from a dispersal event from East Asian *Tacamahaca* species.

### 3.3 Biogeographical analyses

**Figure 4:**
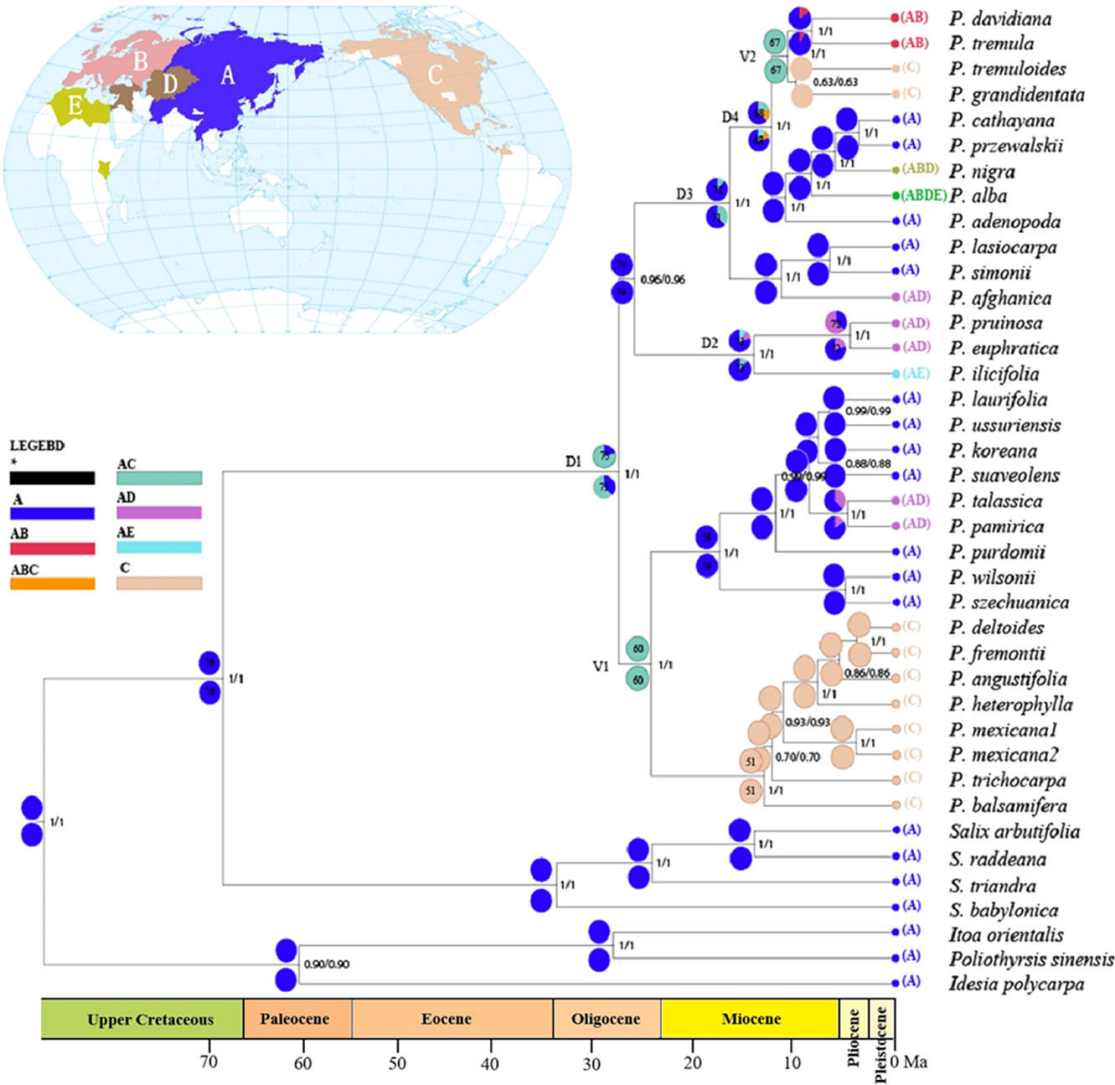
Ancestral area reconstruction for *Populus* based on statistical dispersal-vicariance analysis (S-DIVA) and dispersal-extinction-cladogenesis (DEC) overlaid onto the maximum clade credibility chronogram from BEAST. Current distributions are indicated before the species names. Five areas were defined: **(A)** Asia and neighboring areas, **(B)** Europe, **(C)** North America (including Mexico), **(D)** Center Asia, and **(E)** North Africa; black with an asterisk represents other ancestral ranges. Pie charts at nodes indicate probabilities of alternative ancestral ranges obtained by DIVA (below) and DEC (above). D1–4 and V1–2 indicate dispersal and vicariance events, respectively. Nodes of interests were marked as 1-11 in pie charts.

The results from reconstructions of *Populus’s* ancestral area utilizing the DEC and S-DIVA models were very congruent (**Figure 4**). Multiple potential ancestral distribution patterns were predicted for each node; thus, we concentrated on nodes with the highest relative probabilities. The ancestral reconstruction at node 75 (ancestral node) indicates that the most likely ancestral region of the genus *Populus* is in Asia and adjacent areas (A) with later spread to other areas, and this node was strongly supported with a posterior probability value of 1.00. Throughout the evolution of the genus *Populus*, four dispersal episodes (D1–D4) and two vicariance events (V1–V2) may have occurred. A dispersal event was suggested at node 75 from A to C by ancestral reconstruction at nodes 74 and 60. At node 60, a vicariance event occurred (the most favoured ancestral area was AC at this node). One ancestral event persisted in area A, while the other diversified in area C; a second vicariance event was identified at node 67. The two model (DIVA and DEC) analyses all supported that *Populus* originated from Asia and neighbouring areas and subsequently expanded to Europe, North America and North Africa.

## 4 Discussion

### 4.1 Spatial and temporal patterns of the genus *Populus*

Based on our time estimates (**Figure 3A**) and ancestral area reconstruction (**Figure 4**), the stem age of Salicaceae was approximately 68.67 Ma (95% HPD: 63.73–75.05), which is in line with the oldest known *Populus* leaflet fossil record from East Asia (Guo, 1975; Guo, 1983; Guo, 1985; Tao and Xiong, 1986; **Figure 1**; **Supplementary Table S1**) and coincides with the K-Pg mass extinction boundary at approximately 66 Ma. The transition from the Eocene to the Oligocene was marked by a sudden cooling climate due to the establishment of permanent ice sheets in Antarctica (**Figure 3B**; Zachos et al., 2001). Global climatic change at the Eocene‐Oligocene boundary resulted in increased extinction and turnover of regional floras throughout the globe (Ivany et al., 2000; Retallack et al., 2004; Zanazzi et al., 2007; Sun et al., 2014; Nge et al., 2020). According to our results, the genus *Populus* forms a monophyletic group that separated into three distinct clades, which generally correspond to geographic distributions (**Figure 2B**). The three major branches of the lineages were very short in the phylogenetic tree (**Figure 2A**). Therefore, we speculated that the extant *Populus* species might originated from rapid radiation events in the Oligocene (Liu et al., 2017). During that period, the fluctuating global climates tended to be cooler and drier, the contemporary world’s general landform had already evolved, and severe climate changes were occurring (Tiffney and Manchester, 2001; Zachos et al., 2001). Until the Quaternary glaciations, the ensuing polar ice sheets expanded and retreated multiple times and covered the majority of North America and northern Europe (Zachos et al., 2001). Arid climatic belts also evolved in the core of Eurasia and North America during this time period (Milne and Abbott, 2002). Therefore, the cooling climate likely had a significant impact on the evolution and diversification of contemporary poplar species, which are primarily found in temperate and subtropical parts of the Northern Hemisphere. A previous study of organisms, such as Fagaceae (Manos and Stanford, 2001), *Abies* (Aguirre-Planter et al., 2012) and *Populus* (Du et al., 2015), demonstrated that the extreme climate changes that occurred throughout this time led to the dispersal of populations, the extinction of species, and the formation of new species. The estimated divergence of species in Clade I and Clade II occurred in the late Tertiary at 15.80 Ma (95% HPD: 12.16–22.04) and 17.56 Ma (95% HPD: 13.27–22.63), respectively (**Figure 3A**). During this period, the elevation of the Qinghai-Tibetan Plateau (QTP) drastically changed the terrain of eastern Asia and resulted in remarkable environmental variation, which greatly increased species diversity (Wei et al., 2010). The enormous plateau (Qinghai) and deserts from the north presumably acted as significant physical and ecological barriers that restricted migration and floristic exchange between East Asian species (Clade II) and those of Eurasia. Numerous plant genera, including *Juniperus* (Mao et al., 2010), *Cedrus *(Qiao et al., 2007), and *Helleborus L*. (Sun et al., 2001) have been documented to have a tight biogeographic relationship between the Mediterranean and the QTP.

As during the Palaeocene and Eocene (characterized by hot and humid climates) the boreotropics played a key role in facilitating the intercontinental exchange of tropical biota. However, the disruption that occurred during the late Eocene and early Oligocene, when the climate became cooler and drier, has been shown to have played an important role in forming the disjunct distribution patterns that are observed currently in several vascular plant lineages through vicariance (Baker and Couvreur, 2013; Thomas et al., 2015). The divergence time of current *Populus* species was about 27.24 Ma, which was consistent with the estimation of the destruction time of boreotropic flora. As for the species of *Poulus* species distributed in East Asia and North America, the former region often possesses a greater variety of species than the latter.

The relict branch formed the most recent common ancestor (MRCA) of extant *Populus* species was found in the early Oligocene (27.24 Ma) (**Figure 3A**). The climate during the Oligocene is characterized by climate fluctuations and a long-term cooling tendency (**Figure 3B**). Permanent Antarctic ice sheets lasted until the Late Oligocene, when a warming trend diminished their extent (Zachos et al., 2001). This warm phase reached its maximum extent during the late Mid-Miocene Climatic Optimum (MMCO, 15–17 Ma), which was then immediately followed by a progressive cooling of the temperature and the formation of a significant ice sheet in Antarctica by 10 Ma. (Flower and Kennett, 1995; Hao et al., 2015). These alterations in the Antarctic ice sheets are evidence of a globally significant climate oscillation that caused the BLB to become accessible for trade during a period of warmer temperatures (Tiffney, 1985a; Tiffney, 1985b). Since the Late Cretaceous period, the biological link that connects western North America and eastern Asia has remained mostly unchanged (Wen et al., 2016). Through the NALB and BLB, migration between Eurasia and North America was conceivable during the Cretaceous and Early Tertiary (Manchester et al., 2009). Throughout the majority of the Tertiary, the BLB was a key high-latitude link for plants between eastern Asia and North America (Tiffney, 1985a; Novacek, 1999).

**Figure 5:**
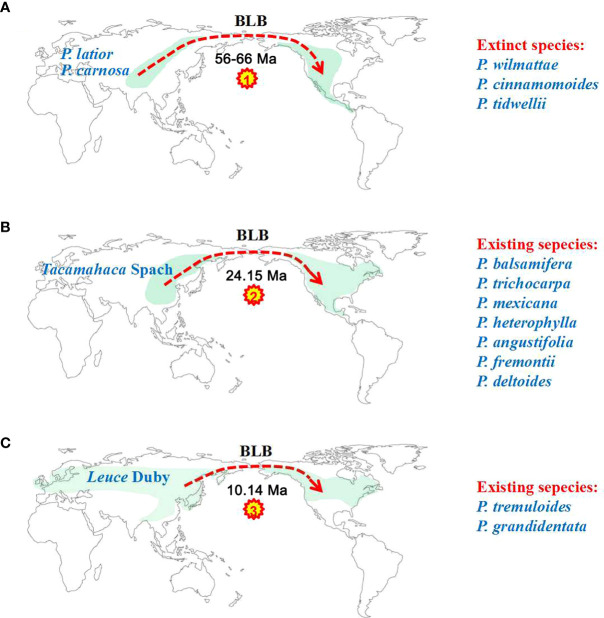
Three colonization events of North America by Eurasian poplar taxa *via* the BLB. **(A)** Descendants of the first colonization event of North America eventually became extinct. **(B)** The second colonization of *Tacamahaca* section species of North America from East Asia produced modern North American *Populus* species. **(C)** The third colonization event of North America formed *P. tremuloides* and *P. grandidentata*.

Our ancestral reconstruction indicates that four dispersal (D1-D4) and two vicariance (V1-V2) events from Asia and neighbouring areas to North America have occurred (**Figure 4**). We found that Eurasian *Populus* species might have colonized the Americas three times through the BLB (**Figure 5**). *Populus* firstly reached North America *via* the BLB and/or NALB during the Paleo (approximately 56 Ma) or Upper Cretaceous (**Figure 5A**). The only remaining genetic traces of ancient species are from chloroplast capture (the chloroplast genome of one species was completely swapped out for that of another) by male plants of the ancestral species of *P. mexicana*, which also became extinct, such as *P. cinnamomoides* (Lesquereux) MacGinitie (MacGinitie, 1969; Eckenwalder, 1980), *P. wilmattae* Cockrell (Manchester et al., 1986), and *P. tidwellii* sp. n. (Manchester et al., 2006). Chloroplast capture is a common phenomenon in biological organisms (Petit et al., 2004). Well-noted examples of chloroplast capture have been reported, such as *Eucalyptus* (Rieseberg and Soltis, 1991), *Helianthus* (Ellis et al., 2008), *Nothofagus* (Acosta and Premoli, 2009), *Lactuca* (Wang et al., 2013) and *Quercus* (Deren et al., 2015). Among these convincing cases, both domestic and wild species are involved, and hybridization between intrageneric and intergeneric species occurs (Tsitrone et al., 2003). Thus, chloroplast capture is not rare and might be a common occurrence in Northern Hemisphere temperate plants. The second colonization was the dispersal of the ancestor species of the *Tacamahaca* section in east Asia during 24.15 Ma (95% HPD: 19.03–29.12; **Figure 5B**), which differentiated into today’s North American *Populus* species (*P. balsamifera*, *P. trichocarpa*, *P. mexicana*, *P. heterophylla*, *P. angustifolia*, *P. fremontii*, and *P. deltoides*). *P. mexicana* may be an ancient hybrid produced by the ancestral species that first dispersed to North America and retreated to Mexico to capture chloroplasts of the ancestors of *P. trichocarpa* and *P. balsamifera* that spread to North America for the second time, and the ancestor species of modern *P. mexicana* became extinct as the climate cooled. The third arrival in North America was the spread of the ancestor of Eurasian aspen at 10.14 Ma (95% HPD: 7.26–12.97), which and differentiated to form present-day *P. tremuloides* and *P. grandidentata* (**Figure 5C**). Both long-distance dispersals across the BLB and vicariance events probably contributed to the patterns of disjunction between eastern Asia and western North America in *Populus*. The resulting disjunctions have previously been inferred in plants (Donoghue and Smith, 2004; Wen et al., 2016) as well as animals (Sanmartín et al., 2001). These patterns of dispersal are mostly consistent with the hypothesis that the majority of the world’s surviving temperate forest flora began in east Asia and spread out over the course of the past 30 Ma (Donoghue and Smith, 2004; Wen et al., 2016). It is believed that the extensive pattern of divergent distributions of temperate forest species among Asia (mainly in East Asia) and North America is at least partially attributable to colonization across the BLB (Wen, 1999). However, another equally likely alternative scenario, given that fossils of the poplars are recorded in North America from the early Eocene, is that the ancestor of the genus *Populus* was once widespread across the Northern Hemisphere (including North America), but then due to higher extinction in Northern America, it was wiped out. Extinction and fragmentation of the original distributional range (vicariance) can occur if the changes caused by abiotic factors such as abrupt climatic or tectonic events are too rapid or large for species to adapt or migrate to more favourable areas (Wiens, 2004). For example, the famous ‘Rand Flora’, a continental-scale biogeographic disjunction pattern that suffered high extinction rates due to successive aridification waves, in northern and central Africa once had a widespread distribution, but now it is fragmented (Sanmartín et al., 2010; Pokorny et al., 2015). In addition, similar examples can be found in the Australian flora (Crisp and Cook, 2007; Nge et al., 2021). This uncertainty in our results may be due to the effects of extinction during *Populus*’*s* evolutionary history. Erroneous inferences about past geographical ranges are more likely when there are inconsistent extinction rates between different regions (Sanmartín and Meseguer, 2016). However, the Eurasian region has relatively higher *Populus* species diversity than North America (Boucher et al., 2003), a large number of *Populus* species living in Asia during the Eocene and Oligocene are well documented (**Supplementary Table S1**), and the less affected historical extinctions may have driven models towards an inference of Asia as an ancestral area. Biogeographic models and analyses based on current species distributions can be misleading due to the extinction of certain lineages (Sanmartín and Meseguer, 2016). Thus, the scenario of Eurasia as an ancestral area followed by long-distance dispersal should be treated with caution, and other alternative scenarios, such as an ancestral distribution that was once more widespread across the Northern Hemisphere (including North America) but destroyed by extinction, should also be considered in biogeographic studies.

### 4.2 The diversification of extant *Populus*

The diversification of the extant *Populus* is largely the result of *in situ* speciation with each of the defined areas during the early Oligocene and following a few independent dispersal and vicariance events at different time periods (**Figures 3**, **4**). Our analyses indicate that the extant *Populus* may experienced three independent North American dispersal events *via* the BLB (**Figure 5**). An examination of the biogeography of a few further Eurasian species, such as Betulaceae (Forest et al., 2005), Rosaceae (Lo et al., 2009), and Sapindaceae (Forest et al., 2001; McClain and Manchester, 2001), indicates similar patterns. The ancestry of today’s poplars can be traced back to the early Oligocene (27.24 Ma), but their current diversity did not emerge until the early Miocene (17.56 Ma) (**Figure 3A**) based on our study. Several other important East Asian angiosperms also diverged about this time: *Castanopsis* (D. Don) Spach (approximately 18.5 Ma; Fagaceae; Xing et al., 2014), *Michelia* L. (approximately 21 Ma; Magnoliaceae; Nie et al., 2008), and Tribus Theeae DC. (approximately 19.4 Ma; Theaceae; Yu et al., 2017), which diverged in the early Miocene. The existence of several comparable fossil taxa on both sides of the Pacific implies that interactions between eastern Asia and North America were possible *via* the BLB from the Cretaceous to the late Neogene (Manchester et al., 2009). The MRCA of extant poplar species most likely survived because it was less affected by glaciers than North America and diverged in the Paratethys region of central Eurasia (mainly in East Asia) based on the Qaidam Basin leaf fossils of *Populus* (Song et al., 2020). Some potential sister groups or all Salicaceae outgroups exist only in Asia (Boucher et al., 2003), and similar patterns have been observed in Betulaceae (Forest et al., 2005), Rosaceae (Lo et al., 2009), and Sapindaceae (Forest et al., 2001; McClain and Manchester, 2001).

Within Clade I, there were two pairs of sister species (*P. tremuloides* and *P. grandidentata*), which were sister to Eurasian species, *P. davidiana* and *P. tremula*, exhibiting a Eurasia–North America temperate disjunct distribution (**Figure 2**). Their divergence time was estimated to be about 10.14 Ma (95% HPD: 7.26–12.97), which indicates that the long-distance dispersal across Eurasia and North America may have occurred through the BLB, resulting in *P. tremuloides* and *P. grandidentata*. This phenomenon is widespread in plants such as Fagaceae (Wen et al., 2016). The BLB broke down from 5.4 to 5.5 Ma (Gladenkov et al., 2002) and linked East Asia and North America before that period. The BLB has played a crucial role in the spread of numerous other species (Feng et al., 2012; Cai et al., 2014). The divergence of a pair of sister species, *P. tremuloides* and *P. grandidentata* (**Figure 3A**), may be related to the continuous decline in the climate on the North American continent 11.8 Ma (Zachos et al., 2001).

Our data suggest that most North American species (Clade III) and East Asian species (Clade II) were grouped together, Clade III split from Clade II at 24.15 Ma (95% HPD: 19.03–29.12), and its divergence time was approximately 12.50 Ma (95% HPD: 7.62–18.93) (**Figure 2**). Therefore, we speculated that dispersal events occurred among western North America and East Asia and that ancestral taxa likely spread to North America *via* the BLB. Clade III did not diversify immediately after it reached North America but only after the climate began to cool. Allopatric speciation and the breakage of biotic links between Tertiary floras of North America and East Asia may have been aided by the drastic climatic shifts that occurred between 15 Ma and the Last Glacial Maximum (Tiffney and Manchester, 2001; Zachos et al., 2001). North American Clade III is monophyletic, but it is divided into three sections (*Aigeiros*, *Leucoides*, *Tacamahaca*). Therefore, the North American lineages may well be a combination of both vicariance and dispersal events in different time periods.

*Turanga* is the basal section, as shown in **Figure 2A**, which accords with the findings of Wang et al. (2020). The *Turanga* section, with only three species, is mainly distributed in central Asia (*P. euphratica* and *P. pruinosa*) and northern Africa (*P. ilicifolia*). In addition, there were no differences in leaf morphology between *P. ilicifolia* and *P. euphratica*, but two sister species, *P. euphratica* and *P. pruinosa* were morphologically distinct. The majority of species in this division grow in tropical or subtropical conditions, showing that the genus *Populus* evolved in warmer environments. During the Oligocene or in the Middle Miocene, northern temperate mesic ancestors once included many Mediterranean species that can withstand drought, though just a few relics can be found in eastern Asia (Wen, 1999; Wen, 2001; Appelhans et al., 2016). These data, together with estimations of divergence time (**Figure 3A**), suggest that low-latitude *Turanga* species are likely relics of the Madrean-Tethyan vegetation zone. *P. ilicifolia* might be an earlier relict of Mediterranean species following its origin from subtropical central Asia and subsequent spread to North Africa, where *P. ilicifolia* and *P. euphratica* arose (13.51 Ma, 95% HPD: 8.53–20.02). *P. pruinosa* diverged from *P. euphratica* at approximately 4.15 Ma (95% HPD: 2.37–8.53). The divergence time of these two species coincides with that of the most recent increase in the QTP (An et al., 2001), suggesting that the uplift of the QTP might have been a major factor driving the origin and diversification of *Populus*. Although the chloroplast datasets analyzed here provide the species-level framework of *Populus*, more evidence for relationships within the genus is still needed. Particularly, nuclear genes evidence are required to explain complex evolutionary history of the genus at future studies, because chloroplast datasets usually show a strong geographic pattern not reflecting of true species relationships. In addition, chloroplast datasets and nuclear datasets, as in most cases, are in conflict which each other.

## 5 Conclusions

Our study applied divergence-time estimations and biogeographic models to investigate the origin and ancestral area of *Populus*, providing a robust framework for further research on the genus. Our study indicates that the present-day disjunction in *Populus* can be explained by *Populus* likely originating in Eurasia and subsequently colonizing other regions, including North America. The climatic change in the Oligocene-Miocene likely drove the diversification of present-day poplar species, the modern North American lineages that may well be a combination of both vicariance and dispersal events from different time periods. An alternative scenario of a once widespread ancestral distribution now erased by mass extinction, similar to the African Rand flora, should also be considered in the biogeographic history of *Populus*. Further studies with dense sampling and more evidence are required to test these hypotheses.

## Data availability statement

The datasets presented in this study can be found in online repositories. The names of the repository/repositories and accession number(s) can be found in the article/**Supplementary Material**.

## Author contributions

JZ and ZW designed the study. XL, SD and QH prepared materials and performed most of the experiments. XL, WW, HL, YZ and YJ performed the data analysis. XL and ZW wrote the manuscript. All authors have read and commented on the paper. All authors contributed to the article and approved the submitted version.
