# Supplementary figures and images for: Origin and evolutionary history of Populus (Salicaceae): Further insights based on time divergence and biogeographic analysis
Source: Front Plant Sci. 2022 Dec 16;13:1031087. doi: 10.3389/fpls.2022.1031087 (PMC9815717; doi:10.3389/fpls.2022.1031087)

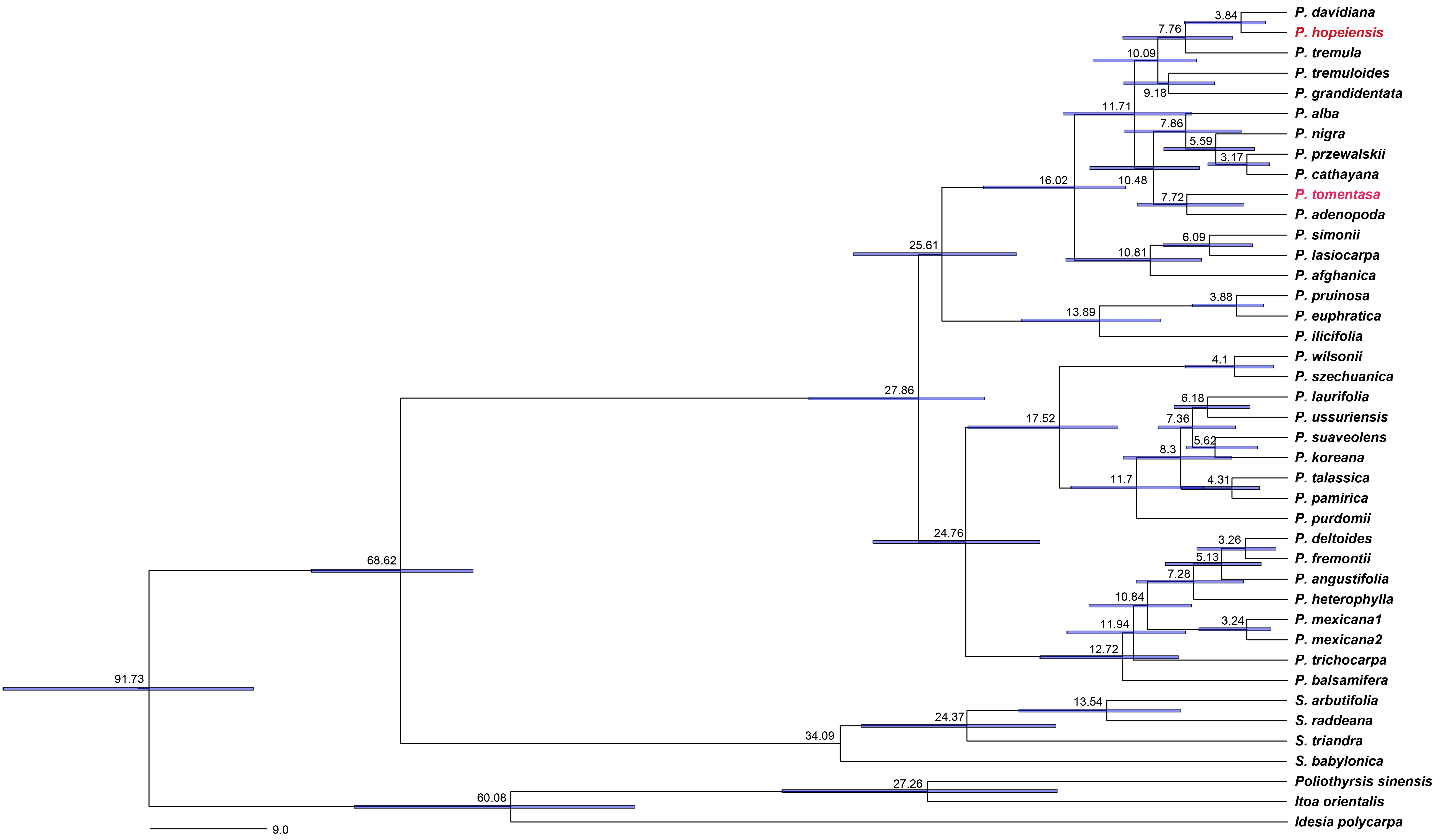

Supplement: Supplementary file 3 [file Image_1.jpeg]
